# Validation of metabolic syndrome using medical records in the SUN cohort

**DOI:** 10.1186/1471-2458-11-867

**Published:** 2011-11-15

**Authors:** Maria Teresa Barrio-Lopez, Maira Bes-Rastrollo, Juan Jose Beunza, Alejandro Fernandez-Montero, Martin Garcia-Lopez, Miguel Angel Martinez-Gonzalez

**Affiliations:** 1 Department of Cardiology and Cardiovascular Surgery, Clínica Universidad de Navarra, Avenida de Pio XII, 36. 31008 Pamplona, Navarra, Spain; 2 Department of Preventive Medicine and Public Health. School of Medicine, University of Navarra, C/Irunlarrea, 1. 31008 Pamplona, Navarra, Spain

## Abstract

**Background:**

The objective of this study was to evaluate the validity of self reported criteria of Metabolic Syndrome (MS) in the SUN (Seguimiento Universidad de Navarra) cohort using their medical records as the gold standard.

**Methods:**

We selected 336 participants and we obtained MS related data according to Adult Treatment Panel III (ATP III) and International Diabetes Federation (IDF). Then we compared information on the self reported diagnosis of MS and MS diagnosed in their medical records. We calculated the proportion of confirmed MS, the proportion of confirmed non-MS and the intraclass correlation coefficients for each component of the MS.

**Results:**

From those 336 selected participants, we obtained sufficient data in 172 participants to confirm or reject MS using ATP III criteria. The proportion of confirmed MS was 91.2% (95% CI: 80.7- 97.1) and the proportion of confirmed non-MS was 92.2% (95% CI: 85.7-96.4) using ATP III criteria. The proportion of confirmed MS using IDF criteria was 100% (95% CI: 87.2-100) and the proportion of confirmed non-MS was 97.1% (95% CI: 85.1-99.9). Kappa Index was 0.82 in the group diagnosed by ATP III criteria and 0.97 in the group diagnosed by IDF criteria. Intraclass correlation coefficients for the different component of MS were: 0.93 (IC 95%:0.91- 0.95) for BMI; 0.96 (IC 95%: 0.93-0.98) for waist circumference; 0.75 (IC 95%: 0.66-0.82) for fasting glucose; 0.50 (IC 95%:0.35-0.639) for HDL cholesterol; 0.78 (IC 95%: 0.70-0.84) for triglycerides; 0.49 (IC 95%:0.34-0.61) for systolic blood pressure and 0.55 (IC 95%: 0.41-0.65) for diastolic blood pressure.

**Conclusions:**

Self-reported MS based on self reported components of the SM in a Spanish cohort of university graduates was sufficiently valid as to be used in epidemiological studies.

## Introduction

Visceral obesity, insulin resistance, high blood pressure, and dyslipidemia are some of the characteristic abnormalities of the metabolic syndrome (MS). The association of these alterations confers a high risk of atherosclerosis and cardiovascular disease, more than the sum of the individual risks [1]. It is estimated that MS, by its consequences, including type 2 diabetes and cardiovascular disease, is the first cause of death in women and the second cause of death in men in our society [2].

A unified definition of MS is needed to detect this entity in the general population [3-5]. The high prevalence (20 to 25% of the world population [6,7]), the dramatic increase over last years [8] and socio-sanitary impact in developed countries is resulting in a global epidemic of MS [9]. In Spain the prevalence of MS varies between 11% and 24%, depending on the different studies [10-14].

An epidemiological questionnaire is a useful tool to study MS in the population. The questionnaire is useful in assessing the characteristics and distribution of MS in the population and then analyzing epidemiological results and drawing conclusions. Sometimes, the only feasible way of studying these populations is to rely on self-reported data of the outcome. As a consequence, it is of the utmost importance to assess the validity of that information. Self-reported diagnosis of MS could not be useful in populations with low cultural level but could be enough valid in a cohort of university graduates.

Our objective was to assess the validity of self-reported diagnosis of MS in a sample of the participants in the Seguimiento Universidad de Navarra (SUN, University of Navarra Follow-up) study, a cohort study in Spain. In this sample we compared the self-reported data in the questionnaire with the data obtained from their medical records as the gold standard.

## Material and methods

The SUN Project is a dynamic cohort of university graduates, recruited and followed up through mailed questionnaires. Beginning in December 1999 [15], the main objective of the study was to assess the association between a Mediterranean dietary pattern and the risk of cardiovascular disease, diabetes, obesity, MS, and other chronic disease. To select the volunteers, all graduates of the University of Navarra, registered nurses from some Spanish provinces, and university graduates from other colleges and associations received a mailed questionnaire and a letter of invitation to participate in the SUN study. A response to the initial questionnaire was considered as informed consent to participate in the study. Then, every 2 years, a follow-up questionnaire is mailed to each participant, gathering information about new medical diagnoses and changes in exposures of interest. Part of this information is self-reported and participants obtain it from their blood test and medical check-ups that they routinely undergo in Spain by their occupational physicians (blood pressure, levels of triglycerides, fasting glucose, high-density lipoprotein, cholesterol, etc.). The questionnaire includes questions about previous and new diagnoses and changes in medications. The diagnosis of MS was not present in the questionnaire. We preferred to ask separately about each of the MS components to avoid an underestimation and to be able to obtain more information (supplementary data file - Additional file 1).

The SUN Study was approved by the Institutional Review Board of the University of Navarra.

### Validation of the study

All participants with MS self reported criteria in the SUN questionnaire and with medical records in the Clinica Universidad de Navarra (CUN) were selected (*n *= 132). Another random sample of 204 participants with no MS criteria in the questionnaire and medical record in the CUN was taken. We calculated this sample size for a single proportion [16] (for a confidence interval of 95%) with a desired precision of the confidence interval of 0.04. Among 336 selected participants, we obtained sufficient data from the medical records to confirm or reject MS in 172 participants. Medical record information and questionnaire information was obtained by a different investigator. Thus, the researcher who obtained information from the medical record (MTB-L) did not know if each participant had reported MS or not. Questionnaires were answered in 2006 and the information from the medical record was obtained between 2005 and 2006.

To conduct the validation we compared the information from the questionnaire with the information from the medical record. The ATP III [3] (*Adult Treatment Panel III*) criteria were applied for the MS classification in the questionnaire and diagnosis in the medical report. In the sensitivity analysis, IDF [4] (*International Diabetes Federation*) criteria were applied too.

For ethical reasons, the identity of the participants was masked. This was the process: a first investigator (C de la F) selected a list with only an identification number, the name and the surname of each participant (self-reported MS criteria were excluded), a second investigator (MTB-L) obtained the medical information about MS from the participants medical record, a third investigator (MB-R) did the analysis between the serf-reported data and the medical history data only with the participant identification number (anonymous).

In this way a) any investigator could not know both information (self-reported and measured information) and the participant identity at the same time b) the investigator who obtained the information from the medical record (MTB-L) was blind with respect to self-reported information; c) the investigator who performed the statistical analysis never knew the participants identity; d) in the moment of answer the questionnaire, participants did not know that we will obtain information from their medical records. Laboratory test results from the medical record and self-reported information were both determined after fasting. Blood pressure, weight and height were measured routinely in the clinic.

### Definition of self-reported MS and confirmed MS

We considered that a participant reported MS if the criteria of MS according to ATP III criteria were present in the questionnaire. On the other hand, we classified a participant as confirmed MS if he or she presented criteria of MS according to ATP III criteria in his/her medical record.

In participants who had normal values of blood pressure, HDL-cholesterol, triglycerides or fasting glycaemia, the medical treatment was considered in order to classify correctly the participants and avoid effect of insulin, lipid-lowering or antihypertensive drugs. Then the same analyses were made using the IDF [4] criteria.

### Statistical analysis

The proportion of subjects with confirmed MS (Positive predictive value, PPV) was calculated as the number of participants who reported MS and this diagnosis was confirmed by the medical history (true positives) divided by the total number of people who reported MS (total positives). In the same way, the proportion of confirmed non MS (Negative predictive value, NPV) was calculated as the number of participants who reported no MS and this diagnosis was confirmed by the medical history (true negatives) divided by the total number of people who reported no MS (total negatives).

The different characteristics (age, sex, body mass index, university career) between the proportion of cases with MS and without confirmed MS were studied using the Fisher's exact test [17].

The coincidence between the self-reported MS and confirmed MS was evaluated by the *Kappa *index. This index was calculated with ATP III and IDF criteria.

To calculate sensitivity and specificity of a self-reported diagnose of MS, the expected distribution of true and false positive and negative cases in the sample were estimated through sampling fractions and observed percentage of confirmed diagnoses to estimate the prevalence of MS in our population. We estimated the prevalence of MS in our population as 20%, which is similar to the prevalence reported by descriptive studies in Spain [10-13]. The Spearman and the Pearson correlation coefficients and intraclass correlation coefficients were calculated for continuous variables.

## Results

We included 57 self-reported MS and 115 self-reported non MS in our analyses. 59.9% were men and 40.1% were women. Mean age was 53.3 years (95% CI: 51.1-56 years), 59 among men and 44 among women. Declared MS increased with age and was higher in men than in women (85% and 15%, respectively). There were no differences in the SM reported between different university degrees. Table 1 shows the main characteristics of the study participants.

**Table 1:** Characteristics of participants in the validation study of the MS according to the ATP-III criteria

	Self-reported MS	Non self-reported MS	Confirmed MS	Confirmed non-MS	Total
	
	n (%)	n (%)	n (%)	n (%)	n (%)
Sex					

Men	46 (80.7%)	57 (49.6%)	51 (83.6%)	52 (46.8%)	103 (59.9%)

Women	11 (19.3%)	58 (50.4%)	10 (16.4%)	59 (53.2%)	69 (40.1%)

Age					

<40 years	5 (8.8%)	41 (35.6%)	4 (6.5%)	42 (37.8%)	46 (26.%)

41-55 years	14 (24.6%)	38 (33%)	12 (19.7%)	40 (36%)	52 (30.2%)

>55 years	38 (66.6%)	36 (31.4%)	45 (73.8%)	29 (26.2%)	74 (43%)

BMI					

<30 kg/m^2^	37 (64.9%)	104 (90.4%)	37 (60.7%)	104 (93.7%)	142 (82%)

≥30 kg/m^2^	20 (35.1%)	11 (9.6%)	24 (39.3%)	7 (6.3%)	30 (18%)

Educational level					

Doctorate/Master 11 (19.3%)		29 (25.2%)	12 (19.7%)	28 (25.2%)	40 (23.3%)

Graduate	22 (38.6%)	38 (33%)	26 (42.6%)	34 (30.6%)	60 (34.9%)

Bachelor	24 (42.1%)	48 (41.7%)	23 (37.7%)	49 (44.2%)	72 (41.9%)

Biomedical	degree				

Yes	13 (22.8%)	50 (43.5%)	14 (22.9%)	49 (44.1%)	63 (36.6%)

No	44 (77.2%)	65 (56.5%)	47 (77.1%)	62 (55.8%)	109 (63.4%)

Total	57	115	61	111	172

*BMI *body mass index

We confirmed 52 (91.22%) of the 57 self-reported MS (95% CI 80.70-97,09%). Among 115 participants who did not report a MS diagnosis in the questionnaires, 106 (92.17%, 95% CI 85.66-96.36%) could be considered non-MS according to our gold standard (Table 2). Table 2 shows diagnosis of MS and validity of self-reported MS according to relevant variables.

**Table 2:** Validity of self-reported MS (Pearson's chi-square) according to relevant variables and ATP-III and IDF criteria

		ATP-III				IDF				
	**%**	**Presence of MS**	**p**	**Absence of MS**	**p**	**%**	**Presence of MS**	**p**	**Absence of MS**	**p**
		**% confirmed (95% CI)**		**% confirmed (95% CI)**			**% confirmed (95% CI)**		**% confirmed (95% CI)**	

Total	100 (*n *= 172)	91.2 (80.7- 97.1)		92.2 (85.7-96.4)		100 (*n *= 62)	100 (87.2- 100)		97.1 (85.1- 99.9)	

Men	59.9	91.3 (79.2-97.6)	<0.00 1	84.2 (72.1- 92.5)	<0.001	59.7	100 (83.2-100)	<0.001	100 (80.5-100)	<0.001

Women	40.1	90.9 (58.7-99.8)		100 (93.8-100)		40.3	100 (59.0-100)		94.4 (72.7-99.8)	

Age (years)										

≤40	26.7	80.0 (28.4-99.5)		100 (91.4-100)		35.5	100 (39.8-100)		100 (81.5-100)	

41-55	30.2	78.6 (49.2- 95.3)	<0.001	97.4 (86.2- 99.9)	<0.001	27.5	100 (54.1-100)	<0.001	100 (71.5-100)	<0.001

>55	43.1	97.4 (86.2-99.9)		77.8 (60.8-89.9)		37	100 (80.5-100)		83.3 (35.9-99.6)	

BMI (kg/m^2^)										

<30	82	89.2 (85.6- 91.9)	<0.001	96.2 (93.6- 96.4)	<0.001	66.1	100 (71.5- 100)	<0.001	100 (88.4- 100)	<0.001

≥30	18	95.0 (93.3- 96.3)		54.5 (23.4-83.2)		33.9	100 (54.1- 100)		80.0 (28.4- 99.4)	

Educational level										

Doctorate/Master	23.2	90.9 (87.9- 93.2)		93.1 (90.8- 94.8)		9.7	100 (54.1- 100)		NA	

Graduate	34.9	95.5 (93.9- 96.6)	<0.001	86.8 (82.6- 90.1)	<0.001	37.1	100 (66.4- 100)	<0.001	100 (76.8- 100)	<0.001

Bachelor	41.9	87.5 (83.4- 90.6)		95.8 (94.4- 96.9)		53.2	100 (73.5- 100)		95.2 (76.2- 99.8)	

Biosanitary degree										

No	63.4	92.5 (79.6- 98.4)	<0.001	87.8 (75.2- 95.4)	<0.001	61.3	100 (83.9- 100)	<0.001	75 (19.4- 99.4)	<0.001

Yes	36.6	84.6 (54.5- 98.1)		94.0 (83.4- 98.7)		38.7	100 (54.1- 100)		100 (81.5- 100)	

*NA *Not applicable. Absence of participants without MS according to IDF criteria in Doctorate/Master group

Based on the assumption that the cohort prevalence of MS was 20%, we estimated a sensitivity of 66% and a specificity of 98% to detect MS using self-reported data. The Kappa index coefficient between serf-reported MS and confirmed MS was 0.82 (95% CI 0.73-0.91%) using ATP III criteria and 0.97 (95% IC: 0.90-1) using IDF criteria.

On the other hand, we analyzed the correlation between component elements of the diagnostic criteria of MS. The best correlation was found for body mass index (BMI). Blood pressure was the variable with the worse correlation. However, all self-reported components of MS had a strong positive correlation according to the available MS components in medical records (Table 3).

**Table 3:** Correlation between self-reported and measured MS variables

Variables	Pearson's r	95% CI	Spearman's Rho	95% CI	Intraclass Correlation Coefficient	95% CI
BMI	0.95	0.93- 0.97	0.91	0.88- 0.93	0.93	0.91-0.95

Waist circumferece	0.96	0.92- 0.98	0.96	0.93- 0.98	0.96	0.93-0.98

Glycaemia	0.74	0.65- 0.81	0.78	0.71- 0.84	0.75	0.66-0.82

HDL	0.54	0.39- 0.66	0.87	0.82- 0.90	0.50	0.35-0.63

Triglycerides	0.79	0.65- 0.88	0.87	0.82- 0.90	0.78	0.70-0.84

SBP	0.52	0.38- 0.63	0.58	0.46- 0.67	0.49	0.34-0.61

DBP	0.55	0.42- 0.66	0.61	0.50- 0.70	0.55	0.41-0.65

*BMI *Body mass index

*SBP *Systolic blood pressure

*DBP *Diastolic blood pressure

*CI *confidence interval

## Discussion

Our findings showed an acceptable degree of confirmation of self-reported diagnoses of MS. Sensitivity was only moderate but specificity and predictive values were very high. In addition, the correlation between the component variables of MS was fair enough to be used in epidemiological studies.

In general, data recorded of MS like a binary expression (presence or absence of MS) has its limitations, and it is better to obtain information of the component elements of the diagnostic criteria of MS (HDL-cholesterol, LDL-cholesterol, fasted glycaemia, blood pressure and waist circumference) to avoid an underestimation. In the SHIELD [18] study (*Study to Help Improve Early evaluation and management of risk factors Leading to Diabetes*) prevalence of self-reported MS was studied in 211.097 participants. SHIELD respondents were asked if they had ever been told by a doctor, nurse or other health professional that they had MS or syndrome X. The proportion of respondents reporting a diagnosis of MS was estimated as the prevalence of MS in SHIELD. Only 0.6% of the total population reported a MS diagnosis. In contrast, NHANES [19] (*n *= 10,780) data using clinical and laboratory criteria indicated a MS prevalence of 25.9% in the adult population. The SHIELD respondents and NHANES participants belong to similar populations according to age, sex and ATP III criteria.

SHIELD study evidenced the lack of knowledge about MS in the general population and especially in high risk patients (obese, diabetic, hypertensive). In these risk populations the self-reported MS was similar to the general population. Therefore in our study we decided to ask for MS criteria to get more information and avoid an underestimation of the true prevalence.

In the SUN cohort we have reported a previous validation of the MS components [20]. In the present study we wanted to validate not only the MS components, but also the MS according to the two more important criteria (IDF and ATP III). We collected continuous data of blood pressure, HDL-cholesterol, triglycerides, weight, height, and waist circumference. From this data MS was defined according with ATP III and IDF criteria.

Positive predictive value (91.2%) and negative predictive value (92.2%) are an evidence of the good validity of self-reported questionnaire to detect the presence or the absence of MS in this population of university graduates.

Correlation between components of MS was good too. As expected, blood pressure was the variable with worse correlation (*r *= 0.44 *p *< 0.001 for systolic blood pressure, *r *= 0.5 *p *< 0.001 for diastolic blood pressure). This phenomenon may be due to higher within individual day-to-day biological variability. The best correlation was observed for weight and height. In the other variables the correlation was good (*r *= 0.68 *p *< 0.001 for fasting glucose, *r *= 0.5 *p *< 0.001 for HDL-cholesterol, *r *= 0.69 *p *< 0.001 for triglyceride, *r *= 0.95, *p *< 0.001 for BMI).

There are several validation studies relating to the waist and hip circumferences [21,22]. They show a sensitivity of 100% and a specificity of 95.7% for men and a sensitivity of 95.1% and a specificity of 97.2% for women to classify in two groups of abdominal obesity when it is compared to the gold standard (measurement made by an expert) [21].

Another study showed a trend towards underestimation of waist circumference in women especially in the group who have a higher obesity abdominal [22]. In our study we found the limitation of poor measurement of waist and hip circumference in clinical practice. However we found a good correlation between self-reported waist circumference and waist circumference from medical records (*r *= 0.96 *p *< 0.001).

About blood pressure, we should take into account that there were occasional large fluctuations due to biological variability. In this context, the participants reported a single measurement and we collected only other measurement from medical history. In no case we had several measurements and in many cases these measurements could be influenced by white coat phenomenon or by medication. This last situation has been taken into account considering as hypertensive those patients diagnosed with hypertension and those taking antihypertensive drugs to avoid misclassification.

Isolated arterial hypertension has been previously validated in a subsample of the SUN cohort with enough validity [23].

We analyzed the differences between MS and no-MS and we observed more MS in men than in women and an increased prevalence with age. No differences were observed according to university degree. It is possible that in the SUN cohort there are less MS incidence than in the general Spanish population because it is a young cohort (mean age was 37.5 years).

We only could diagnose or rule out MS with information found in medical records in 172 of the 336 participants initially selected. This was a possible limitation of our study because of the existence of a potential selection bias. However, the sociodemographic characteristics and self-reported averaged values for the components of MS were similar in both groups with no statistically significant differences.

We accept that the sample was not representative of the general population because it is a cohort of university graduates and this could be considered as a limitation in our study. It is possible that university graduates have better knowledge about MS and about health in general that general population. However, this could be considered also as a strength of the study because our aim was to validate self-reported diagnosis of MS and its components among highly educated subjects (i.e. in this cohort). This would allow us to use the self-reported information from these highly educated participants to conduct studies of association between various risk factors and this entity. In this context, we should note that it is not necessary for the sample to be representative, in the statistical sense of random sampling, of the general population. Indeed, the sample of this 336 selected participants should be representative of all the SUN cohort participants. This situation contributes to increase the internal validity of our results because we are obtaining self-reported information of high quality. Moreover, uniform education of the cohort reduces the potential confounding effect by socioeconomic factors. On the other hand, a strong point of our design is that participants were unaware that we could get information from their medical records after answering their questionnaire. It thus prevents them to overstate or be artificially more precise in their reported data.

## Conclusions

Self-reported MS based on self-reported components of the SM in a Spanish cohort of university graduates was sufficiently valid as to be used in epidemiological studies.

## Abbreviations

MS: metabolic syndrome; CI: confidence interval; SUN: Seguimiento Universidad de Navarra, University of Navarra Follow-up Study; CUN: Clinica Universidad de Navarra; BMI: body mass index; SBP: Systolic blood pressure; DBP: Diastolic blood pressure.

## Competing interests

The authors declare that they have no competing interests.

## Authors' contributions

MTB-L participated in the study design, the acquisition of data, the analyses, and the first drafting of the manuscript. MB-R participated in the acquisition of data, the analyses, and in the interpretation of results. JJB, AF-M and MG-L participated in the study design and in the interpretation of data. MAM have made substantial contributions to conception and design of the study, participated in the statistical analysis and the interpretation of data. All authors participated have revised the manuscript for important intellectual content and read and approved the final manuscript.

## Pre-publication history

The pre-publication history for this paper can be accessed here:

http://www.biomedcentral.com/1471-2458/11/867/prepub

## Supplementary Material

Additional file 1 supplementary data file Click here for file
