# Supplementary material for: Validation of metabolic syndrome using medical records in the SUN cohort
Source: BMC Public Health. 2011 Nov 15;11:867. doi: 10.1186/1471-2458-11-867 (PMC3296781; doi:10.1186/1471-2458-11-867)

Appendix 1. Questionnaire used to define Metabolic Syndrome and instructions to measure waist and hip circunferences

Por favor, indica a continuación los datos de las últimas mediciones que te hayan hecho y que tenga menos de 2 años de antigüedad:

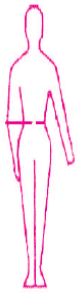

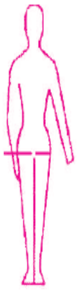

Perímetro cintura (Cm.)	Perímetro cadera (Cm.)	TENSIÓN ARTERIAL (Mm de Hg) Alta (sistólica)	TENSIÓN ARTERIAL (Mm de Hg) Baja (diastólica)	AZUCAR Glucemia basal (mg/dl) Glucosa	COLESTEROL (mg/dl) Total	COLESTEROL (mg/dl) LDL	COLESTEROL (mg/dl) HDL	COLESTEROL (mg/dl) Triglicéridos	Peso actual (Kg.)	Talla (Cm.)
0 0 0	0 0 0	0 0 0	0 0 0	0 0 0	0 0 0	0 0 0	0 0 0	0 0 0	0 0 0	0 0 0
1 1 1	1 1 1	1 1 1	1 1 1	1 1 1	1 1 1	1 1 1	1 1 1	1 1 1	1 1 1	1 1 1
2 2	2 2	2 2 2	2 2 2	2 2 2	2 2 2	2 2 2	2 2	2 2 2	2 2	2 2 2
3 3	3 3	3 3	3 3	3 3 3	3 3 3	3 3 3	3 3	3 3 3	3 3	3 3
4 4	4 4	4 4	4 4	4 4 4	4 4 4	4 4 4	4 4	4 4 4	4 4	4 4
5 5	5 5	5 5	5 5	5 5	5 5	5 5	5 5	5 5	5 5	5 5
6 6	6 6	6 6	6 6	6 6	6 6	6 6	6 6	6 6	6 6	6 6
7 7	7 7	7 7	7 7	7 7	7 7	7 7	7 7	7 7	7 7	7 7
8 8	8 8	8 8	8 8	8 8	8 8	8 8	8 8	8 8	8 8	8 8
9 9	9 9	9 9	9 9	9 9	9 9	9 9	9 9	9 9	9 9	9 9

SUMCO 00000-05 (Ref.)

¡MUCHAS GRACIAS POR TU VALIOSA COLABORACIÓN!

¿CÓMO SE DEBE MEDIR EL PERÍMETRO DE LA CINTURA?

-Póngase de pie y relaje el abdomen
-Mida el contorno del talle natural tomado entre la parte superior del hueso de la cadera (crestas iliacas) y la costilla inferior.

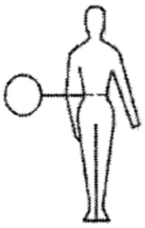

MAL

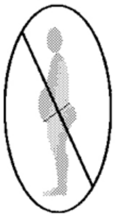

BIEN

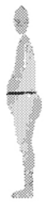

¿Y EL DE LA CADERA?

- En la misma posición mida el contorno horizontal tomado alrededor de las nalgas y al nivel de la circunferencia máxima.

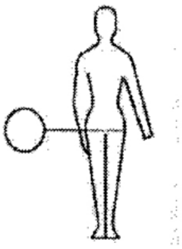

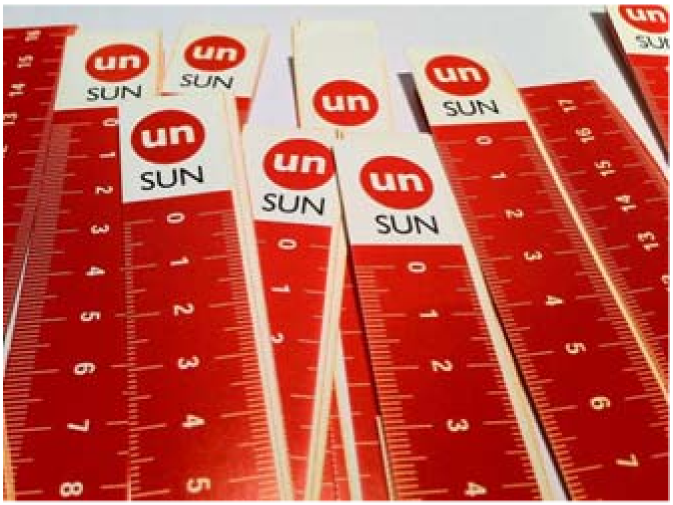

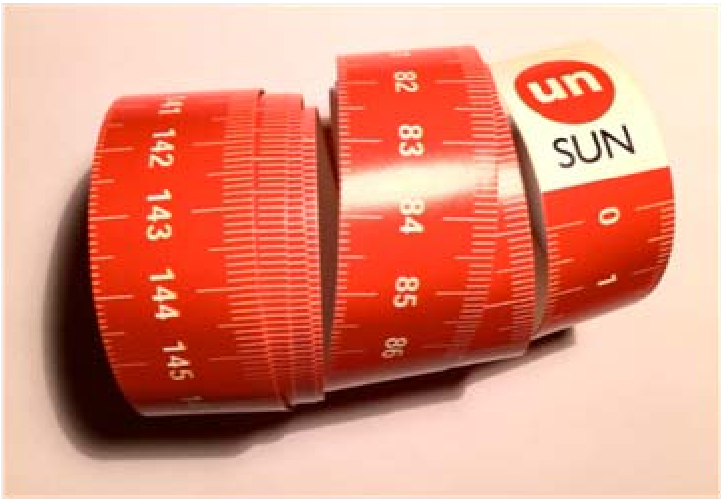

Please, indicate the data of your last measurements within a period of less than 2 years:

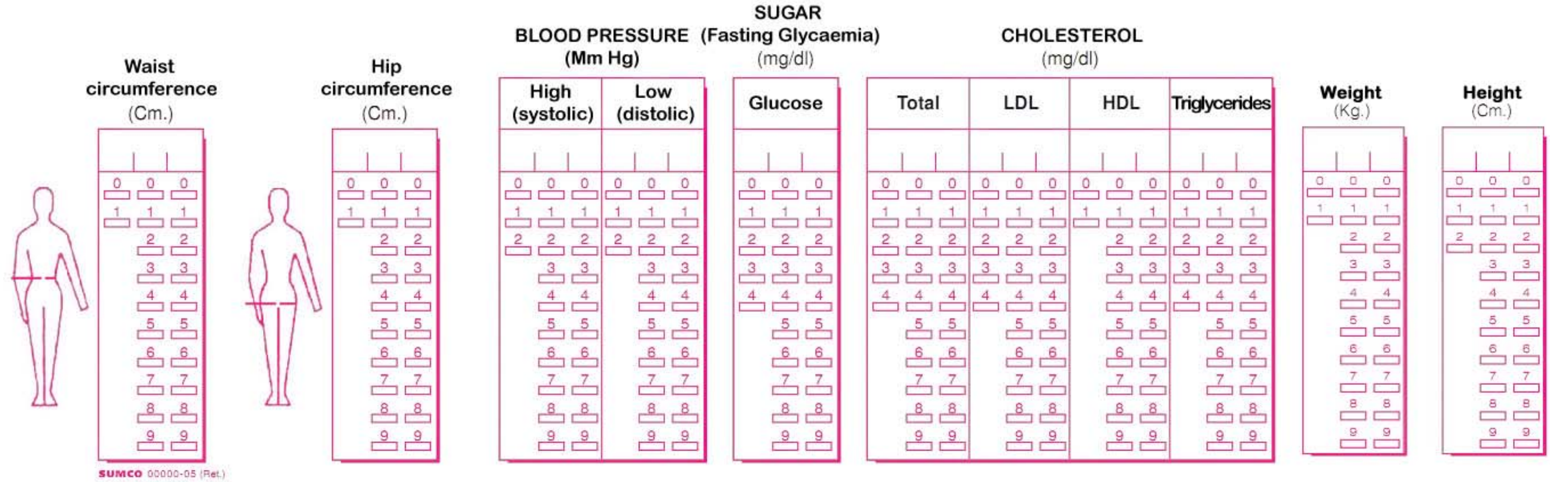

THANK YOU VERY MUCH FOR YOUR VALUABLE COLABORATION!

¿HOW WAIST CIRCUMFERENCE SHOULD BE MEASURED?

-Stand up and relax the abdomen.
-Measure around your high hip bone (iliac crests) and the lower rib.

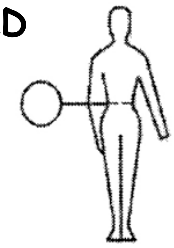

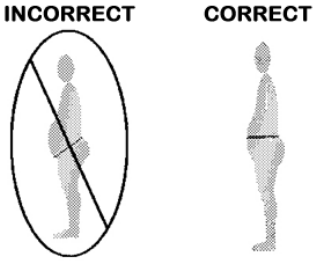

¿AND THE HIP?

-In the same position measure around your largest circumference between your waist and your thighs.

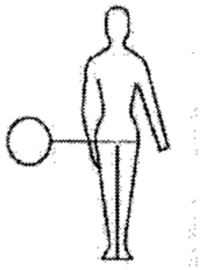

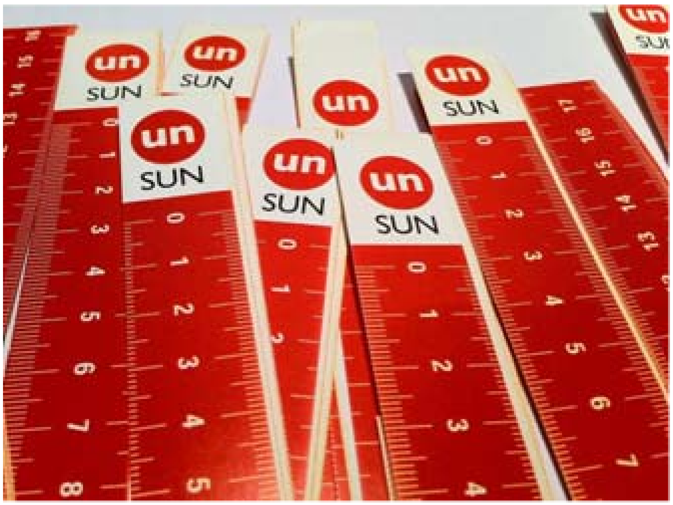

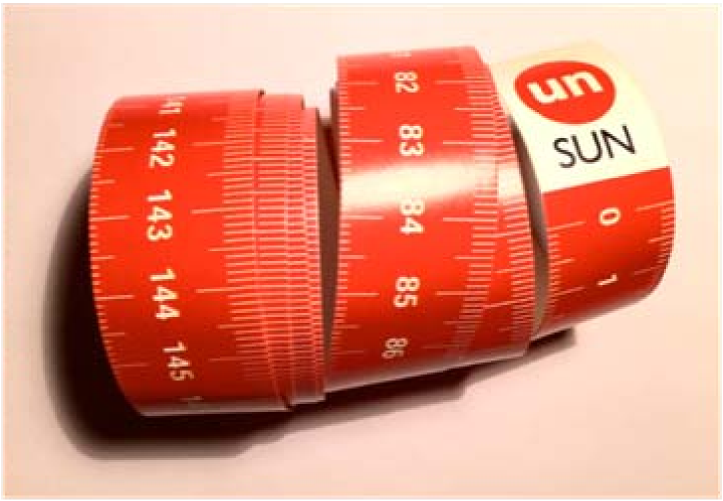

Supplement: Additional file 1 — supplementary data file [file 1471-2458-11-867-S1.PDF]
